# Experimental Measurements for the Effect of Dilution Procedure in Blood Esterases as Animals Biomarker for Exposure to OP Compounds

**DOI:** 10.1155/2014/451982

**Published:** 2014-04-22

**Authors:** Kasim Sakran Abass

**Affiliations:** Department of Basic Nursing Sciences, College of Nursing, University of Kirkuk, Kirkuk, Iraq

## Abstract

Organophosphate compounds can bind to carboxylesterase, which may lower the concentration of organophosphate pesticides at the target site enzyme, cholinesterase. It is unclear from the literature whether it is the carboxylesterase affinity for the organophosphate and/or the number of carboxylesterase molecules that is the dominant factor in determining the protective potential of carboxylesterase. The fundamental dilutions and kinetic effects of esterase enzyme are still poorly understood. This study aims to confirm and extend our current knowledge about the effects of dilutions on esterases activities in the blood for birds with respect to protecting the enzyme from organophosphate inhibition. There was significantly higher esterases activities in dilution 1 : 10 in the all blood samples from quail, duck, and chick compared to other dilutions (1 : 5, 1 : 15, 1 : 20, and 1 : 25) in all cases. Furthermore, our results also pointed to the importance of estimating different dilutions effects prior to using in birds as biomarker tools of environmental exposure. Concentration-inhibition curves were determined for the inhibitor in the presence of dilutions 1 : 5, 1 : 10, plus 1 : 15 (to stimulate carboxylesterase). Point estimates (concentrations calculated to produce 20, 50, and 80% inhibition) were compared across conditions and served as a measure of esterase-mediated detoxification. Results with well-known inhibitors (malathion) were in agreement with the literature, serving to support the use of this assay. Among the thiol-esters dilution 1 : 5 was observed to have the highest specificity constant (*k*_cat_/*K*_*m*_), and the *K*_*m*_ and *k*_cat_ values were 176 *μ*M and 16,765 s^−1^, respectively, for S-phenyl thioacetate ester, while detected in dilution 1 : 15 was the lowest specificity constant (*k*_cat_/*K*_*m*_), and the *K*_*m*_ and *k*_cat_ values were 943 *μ*M and 1154 s^−1^, respectively, for acetylthiocholine iodide ester.

## 1. Introduction

An esterase is a hydrolase enzyme that splits esters into an acid and an alcohol in a chemical reaction with water called hydrolysis [1, 2]. Carboxylesterase (CbE)/cholinesterase (ChE) family members are under esterase enzyme and are responsible for controlling the nerve impulse, detoxification, and various developmental functions and are a major target of pesticides and chemical warfare agents [2–4]. Comparative structural analysis of these enzymes is thus important [2, 5, 6]. Organophosphates (OP) bind strongly and largely irreversibly to both ChE in cholinergic synapses and to CbE, which is found in blood and tissues [7–9]. The reaction with the enzyme is the key to the considerable number of pharmacological actions possessed by OP, although other biochemical properties of these compounds have recently been recognised as important [10]. OP compounds are a group of artificially synthesized substances used in birds to control pests and to enhance agricultural production [11, 12]. However, OP are used as insecticides, acaricides, and chemical agents and share a common neurotoxic mechanism of action [13]. The biochemical alterations leading to many of the deleterious effects have been studied in neuronal cell lines; however, nonneuronal toxic effects of OP are far less well characterized* in vitro* [12, 14]. At present, many hundreds of thousands of tons of OP compounds are used in this way throughout the world. Apart from the interest in OP as military weapons, the past thirty years have seen an unprecedented growth in their use as insecticides, stimulating the production of certain compounds such as malathion which are degraded by higher organisms while remaining toxic to arthropods [12]. Generally, the cause of death in severe OP intoxication is a combination of several factors, which are (a) circulatory effects by decreased cardiac output with bradycardia and peripheral vascular effects; and (b) central nervous system by generalised cortical activity causing convulsions which aggravate the cardiovascular system [15, 16]. Consequently, more efficient processes, such as chemical oxidation, are needed to remove OP during consumption of food production [6, 17, 18]. Malathion is an organophosphate parasympathomimetic that binds irreversibly to esterase enzyme. It is converted to malaoxon by the multifunction oxidases in mammals and insects or undergoes hydrolysis of one of the CH_3_O–P bonds [19–21]. Deactivation may also occur by hydrolysis of the P–S linkage. However, very few studies are focused on the dilution of malathion in the birds; accordingly, this paper was aimed to investigate the effects of dilution on CbE and ChE activities in the blood from birds used for human consumption. A further aim was to indicate the value of maximal inhibitory concentrations and different kinetic effects of malathion in different dilutions as a biochemical biomarker of exposure to pesticides toxicology.

## 2. Materials and Methods

### 2.1. Chemicals

CbE substrate, S-phenyl thioacetate (PSA), 98% purity; ChE substrate, acetylthiocholine iodide (AcTChI), 98% purity; and 5 5′-dithiobis(2-nitrobenzoic acid) (DTNB) were supplied by the Sigma Chemical Company. Malathion [S-1,2-bis(ethoxycarbonyl) ethyl O,O-dimethyl phosphorodithioate] was obtained from G.L. Industries (E) Ltd., Guwahati, India. All other reagents and solvents used in this paper were of analytical grade.

### 2.2. Sample Collection

Blood samples from healthy quail, duck, and chick from local markets in Kirkuk were used in this paper. They were maintained in batches of 3–15 birds in cages with dimensions of 75 × 75 × 75 cm in a room with constant lighting at a temperature of 25–35°C and relative humidity was between (45–50%), which was controlled by electric heaters. The floor litter consisted of wood shavings; water and feed were available ad libitum. Blood samples were from male and female of white and brown,* Japanese quail*; Pekin duck,* Anas platyrhynchos*; and chicks (Ross 308,* Gallus gallus*) ranging in age from 2 to 4 weeks. The Scientific Committee of the College of Nursing at the University of Kirkuk (Iraq) has approved the present study. Experiments detected with institutional regulations addressing animal use and good attention and care 6 were given to the birds used in this work.

### 2.3. Sample Preparation

To obtain plasma, 1 mL blood samples were added to anticoagulant (EDTA, 7.2 mg, final concentration 0.18%) in 5 mL centrifuge tubes. Plasma was separated by centrifugation at 5000 g for 10 min [2]. To obtain serum, blood samples were allowed to clot for at least 2 h at 25°C, after which they were centrifuged at 5000 g for 10 min. The erythrocytes were washed three times with two volumes of phosphate buffer (0.1 M, pH 7.4), centrifuged as described above between washes. Next, the packed erythrocytes diluted in 20 volumes of hypotonic sodium phosphate buffer (6.7 mM, pH 7.4) to facilitate haemolysis followed by centrifugation at 5000 g for 10 min. The supernatant was removed and the pellet resuspended in hypotonic phosphate buffer.

### 2.4. Enzyme Measurement

Esterase enzyme activity was determined at room temperature 25°C by the Ellman method [22], using thioacetate (PSA substrate) for measuring CbE activity or by using thiocholine (AcTChI substrate) for measuring ChE activity [2, 23]. Subsequent combination of thioacetate or thiocholine derivatives with DTNB forms the yellow anion 5-thio-2-nitrobenzoic acid, which absorbs strongly at 410 nm [24]. Substrate solutions (2 mM for PSA, while 1 mM for AcTChI) were prepared and used on the same day and kept on ice during use. The blood samples were diluted with phosphate buffer (0.1 M, pH 8) with a 1 : 5, 10, 15, 20, and 25 ratios (*w/v*) of parts of buffer. All measurements in this paper were carried out in duplicate. Specific inhibitor incubation of the samples with 0.1 mM final concentration of* bis(p-nitrophenyl)phosphate* in case of measuring of CbE, while samples incubated with 4 mM final concentration of tetraisopropyl pyrophosphoramide in case of measuring of ChE.

### 2.5. *In Vitro* Exposure to OP Compound

For the measurement of maximal inhibitory concentrations (IC_20,_ IC_50,_ and IC_80_) blood samples were inhibited for 30 min at room temperature 25°C with appropriate concentration malathion compound, depending on preliminary range finding tests [2, 25]. Controls were incubated with phosphate buffer pH 8.0 included. Then the enzyme activity was determined as described in above section. Then the data were fitted with nonlinear regression analysis using a single exponential decay by SigmaPlot 11 (Systat software, Inc.). The Michaelis constant (*K*_*m*_) and turnover number (*k*_cat_) were determined according to Abass [2].

### 2.6. Statistical Analysis

The statistical analysis was performed using SigmaPlot version 11. Differences among groups were determined by one-way analysis of variance (ANOVA) followed by Fisher's LSD test. All results are presented as mean ± SE and significance is accepted at *P* < 0.05.

## 3. Results 

### 3.1. Determination of Blood Esterase in Birds by the Enzyme-Dilution Method

**Figure 1:**
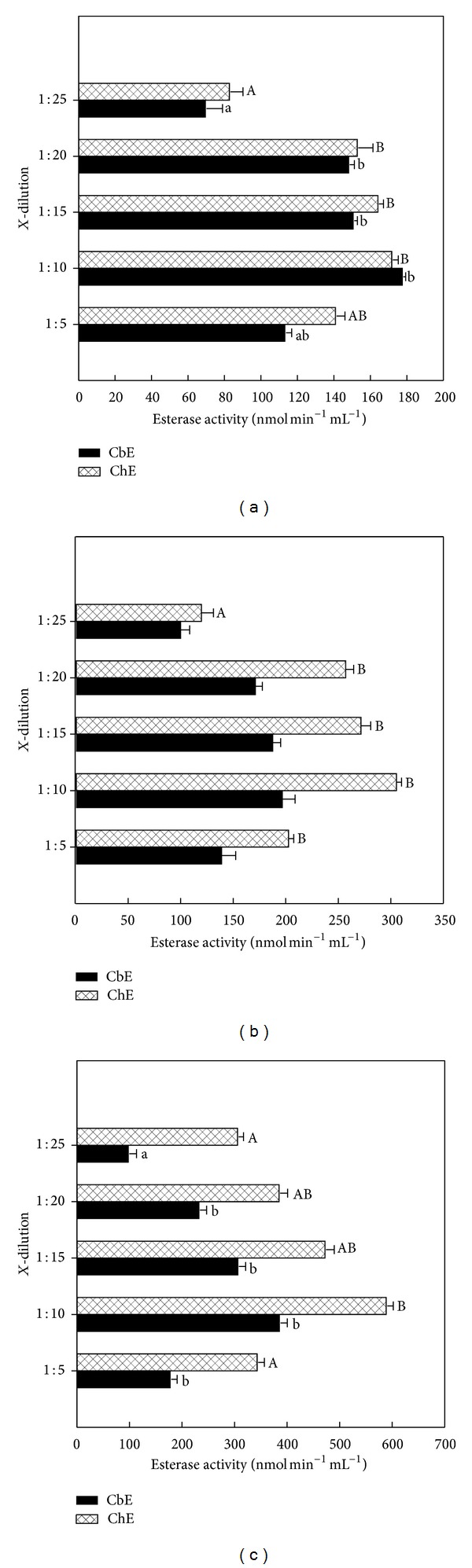
Esterase enzyme activities in plasma diluted for quail (a), duck (b), and chick (c). ^A, B^ denote significantly different between mean ChE with different dilutions (analysis of variance (ANOVA), *P* < 0.05). ^a, b^ denote significantly different between mean CbE with different dilutions (analysis of variance (ANOVA), *P* < 0.05). Each experiment is performed in duplicate (*n* = 5 in each bird).

**Figure 2:**
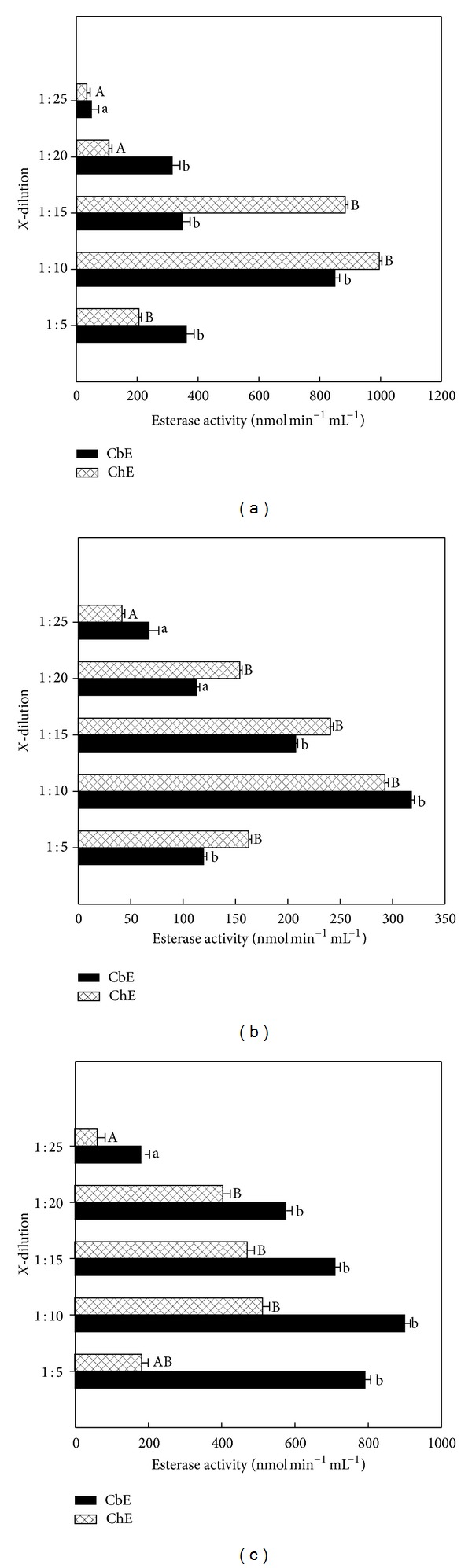
Esterase enzyme activities in erythrocyte diluted for quail (a), duck (b), and chick (c). Key to the figures is listed under Figure 1.

**Figure 3:**
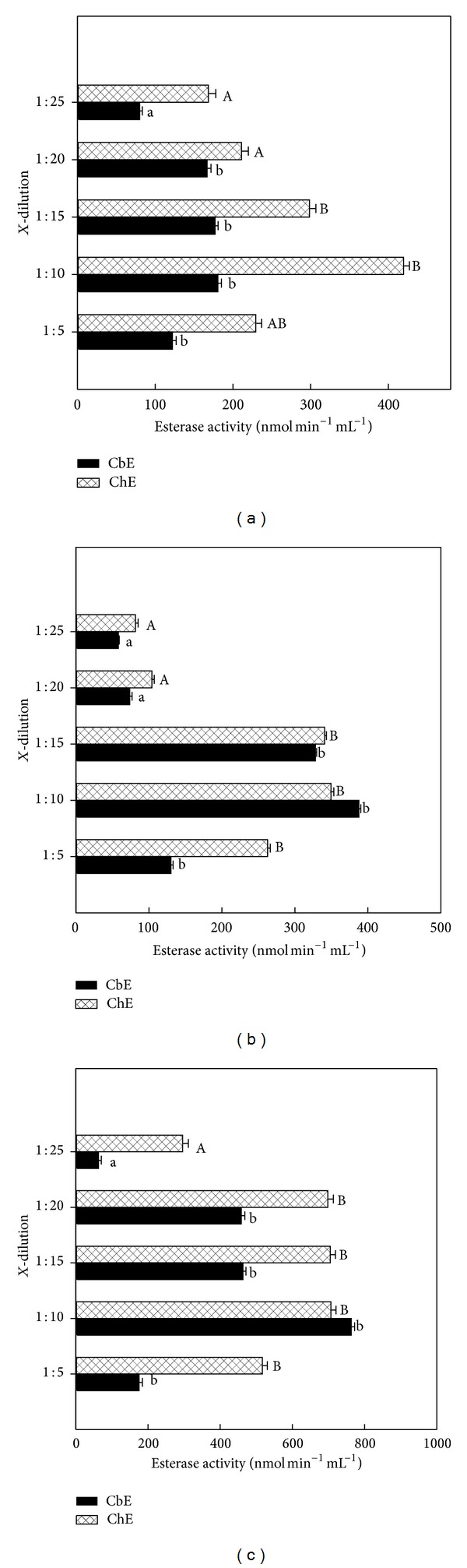
Esterase enzyme activities in serum for quail (a), duck (b), and chick (c). Key to the figures is listed under Figure 1.

The effect of dilutions on CbE and ChE activities was determined in serum, plasma, and erythrocyte for quail, duck, and chick as described in above section of Materials and Methods (Figures 1–3). CbE and ChE activities in dilution 1 : 10 observed highest activity in the plasma, serum, and erythrocyte for quail, duck, and chick (Figures 1–3). It was found that plasma CbE was significant (*P* < 0.05) in dilution 1 : 25 among other dilutions for quail and chick (Figures 1(a) and 1(c)). Blood plasma from the ChE activity in blood from the plasma was significantly different (*P* < 0.05) in dilution 1 : 25 among other dilutions for quail and duck (Figures 1(a) and 1(b)) and in dilution 1 : 10 among other dilutions for chick (Figure 1(c)). The plasma activities of CbE ranged between 70.1 and 178.1 nmol min^−1^ mL^−1^ for quail, between 99.9 and 196.7 nmol min^−1^ mL^−1^ for duck, and between 99.6 and 387.5 nmol min^−1^ mL^−1^ for chick samples across different dilutions, while for plasma ChE ranged between 83.3 and 172.3 nmol min^−1^ mL^−1^ for quail, between 119.7 and 305.3 nmol min^−1^ mL^−1^ for duck, and between 307.7 and 590.7 nmol min^−1^ mL^−1^ for chick samples across different dilutions (Figures 1(a)–1(c)). There was significance (*P* < 0.05) in serum CbE within dilution 1 : 25 among other dilutions used for quail and chick (Figures 2(a) and 2(c)), while serum CbE was not significant (*P* > 0.05) between dilution 1 : 20 and dilution 1 : 25 for duck (Figure 2(b)). ChE activity was a significantly different (*P* < 0.05) between dilution 1 : 10 and dilution 1 : 15 with other dilutions used for quail (Figure 2(a)), while in duck it was seen as not significant (*P* > 0.05) between dilution 1 : 20 and dilution 1 : 25 (Figure 2(b)), whereas for chick was significant (*P* < 0.05) between dilution 1 : 25 among other dilutions (Figure 2(c)). The serum activities of CbE ranged between 80.2 and 180.6 nmol min^−1^ mL^−1^ for quail, between 58.1 and 387.6 nmol min^−1^ mL^−1^ for duck, and between 62.9 and 463.1 nmol min^−1^ mL^−1^ for chick samples across different dilutions, whereas for serum ChE ranged between 211.1 and 420.3 nmol min^−1^ mL^−1^ for quail, between 82.3 and 350.2 nmol min^−1^ mL^−1^ for duck, and between 297.3 and 708.4 nmol min^−1^ mL^−1^ for chick samples across different dilutions (Figures 2(a)–2(c)). The enzyme activity in the serum blood at the higher dilution ratio declined faster than that of samples at lower dilution ratio.

**Figure 4:**
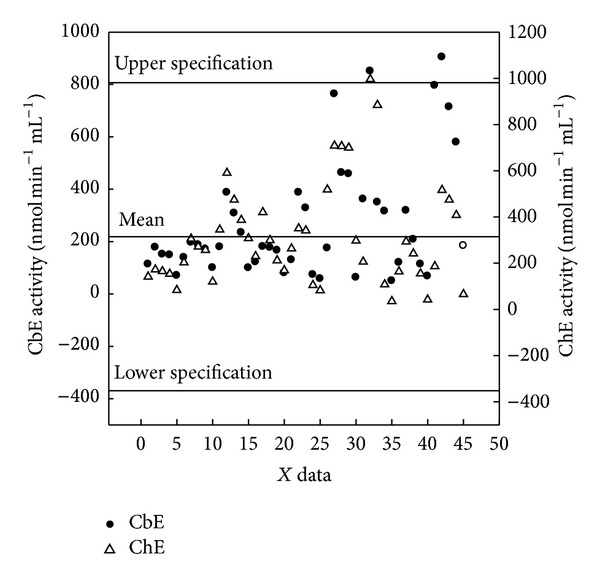
Distribution of the individual values of CbE and ChE activities in all tested birds (quail, duck, and chick) and blood samples (plasma, serum, and erythrocyte) in dilutions (1 : 5, 1 : 10, 1 : 15, 1 : 20, and 1 : 25).

**Figure 5:**
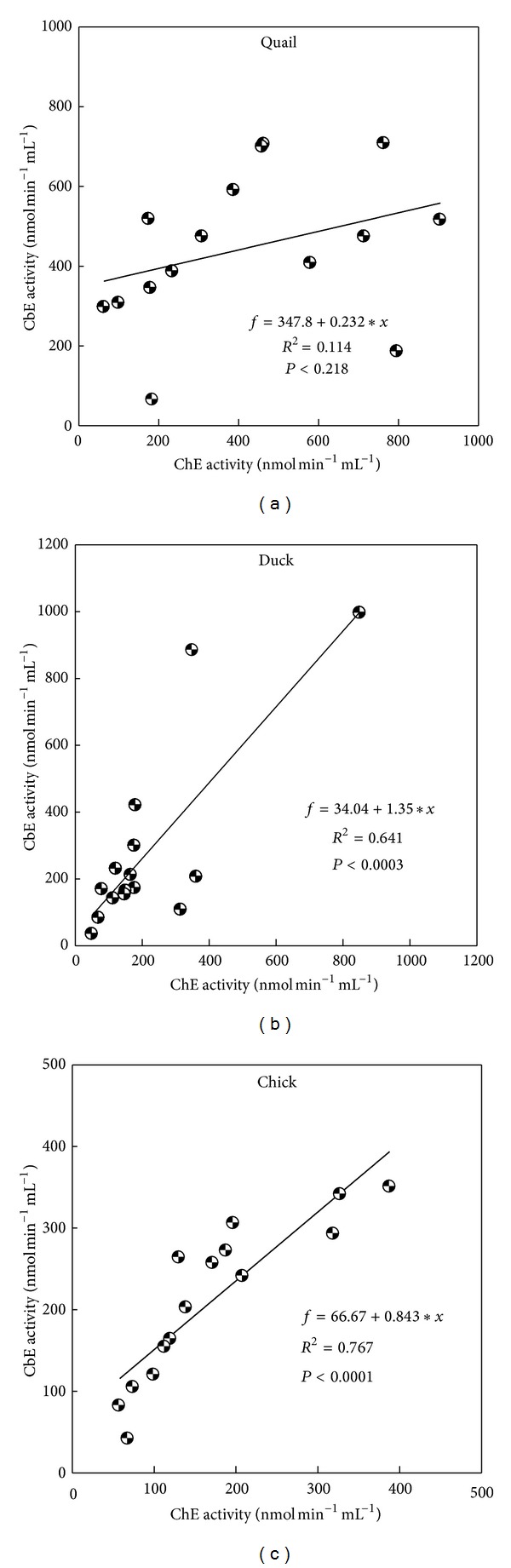
Regression analyses between CbE and ChE activities in all tested blood contents from quail, duck, and chick across different dilutions (1 : 5, 1 : 10, 1 : 15, 1 : 20, and 1 : 25).

**Figure 6:**
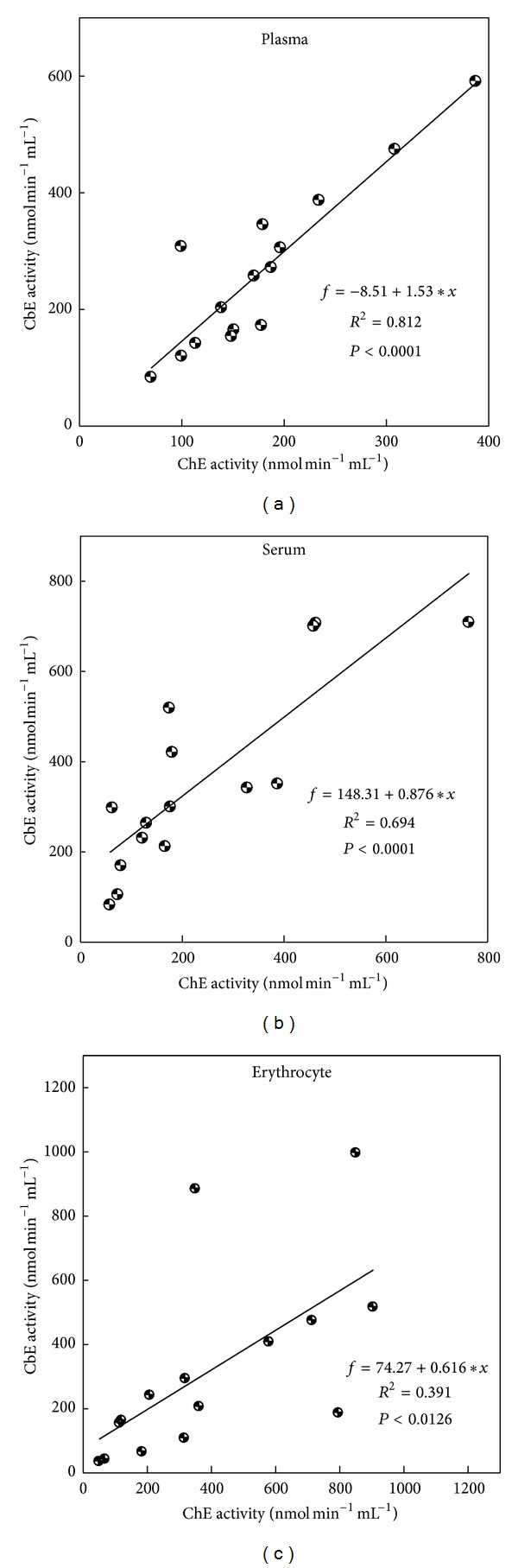
Regression analyses between CbE and ChE activities in all tested birds for plasma, serum, and erythrocyte across different dilutions (1 : 5, 1 : 10, 1 : 15, 1 : 20, and 1 : 25).

**Figure 7:**
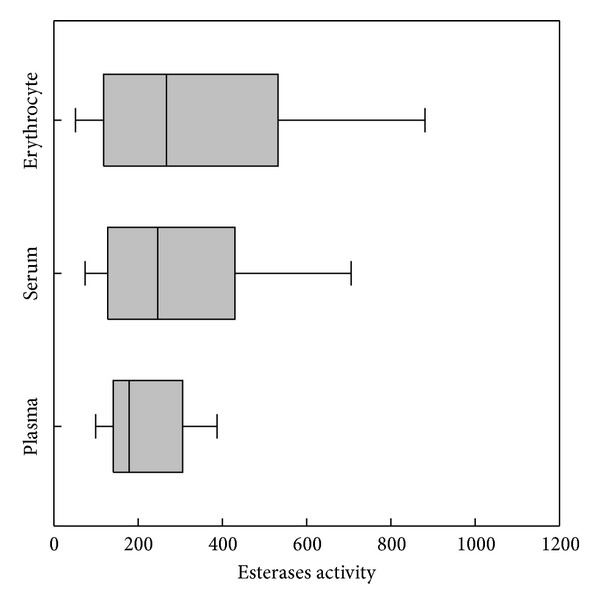
Box plots of the range esterase enzyme activity reported to be taken in different dilutions (1 : 5, 1 : 10, 1 : 15, 1 : 20, and 1 : 25).

Blood from erythrocyte was seen significant (*P* < 0.05) within dilution 1 : 25 among other dilutions for quail CbE and chick ChE (Figures 3(a) and 3(c)) and was seen not significant (*P* > 0.05) between dilution 1 : 20 and dilution 1 : 20 for duck (Figure 3(b)), while there were significant (*P* < 0.05) differences within dilution 1 : 25 among other dilutions used for chick CbE and ChE (Figure 3). ChE was seen not significant (*P* > 0.05) between dilution 1 : 20 and dilution 1 : 25 for quail (Figure 3(a)); erythrocyte activities of CbE ranged between 50.1 and 850.6 nmol min^−1^ mL^−1^ for quail, between 68.4 and 318.8 nmol min^−1^ mL^−1^ for duck, and between 184.2 and 904.7 nmol min^−1^ mL^−1^ for chick samples across different dilutions, whereas for erythrocyte ChE ranged between 35.3 and 996.4 nmol min^−1^ mL^−1^ for quail, between 42.3 and 293.5 nmol min^−1^ mL^−1^ for duck, and between 65.4 and 516.4 nmol min^−1^ mL^−1^ for chick samples across different dilutions (Figures 3(a)–3(c)). Overall study, the dilution 1 : 25 gave a lowest enzymatic activity in both CbE and ChE (Figures 1–3). The distribution of the mean individuals values of esterase enzyme activity in all birds and blood contents in different dilutions was detected the highest esterase activities in ChE (318.7 nmol min^−1^ mL^−1^) compared to CbE (214.4 nmol min^−1^ mL^−1^) (Figure 4). In all cases (quail, duck, and chick used five dilutions), the linear regression found between CbE and ChE was seen to be *R* ^2^ = 0.114, *P* < 0.218 for quail; *R* ^2^ = 0.641, *P* < 0.0003 for duck; and *R* ^2^ = 0.767, *P* < 0.0007 for chick (Figure 5). In addition, the author found a linear regression between all tested blood samples (*R* ^2^ = 0.812, *P* < 0.0001 for plasma; *R* ^2^ = 0.694, *P* < 0.0001 for serum; and*R* ^2^ = 0.391, *P* < 0.0126 for erythrocyte) (Figure 6). The author found blood dilution only with erythrocyte detects in higher activities of CbE and ChE than plasma and serum (Figure 7). Pearson correlation coefficient (*r*) calculated to measure the degree of relationship between CbE and ChE in the plasma, serum, and erythrocyte for testing birds was observed significantly in plasma, serum, and erythrocyte with dilution 1 : 10 for quail and chick, in addition to a significant effect with dilution 1 : 20 for serum of quail and duck (Table 1).

### 3.2. Kinetic Dilution Methodology for the OP Compound

**Figure 8:**
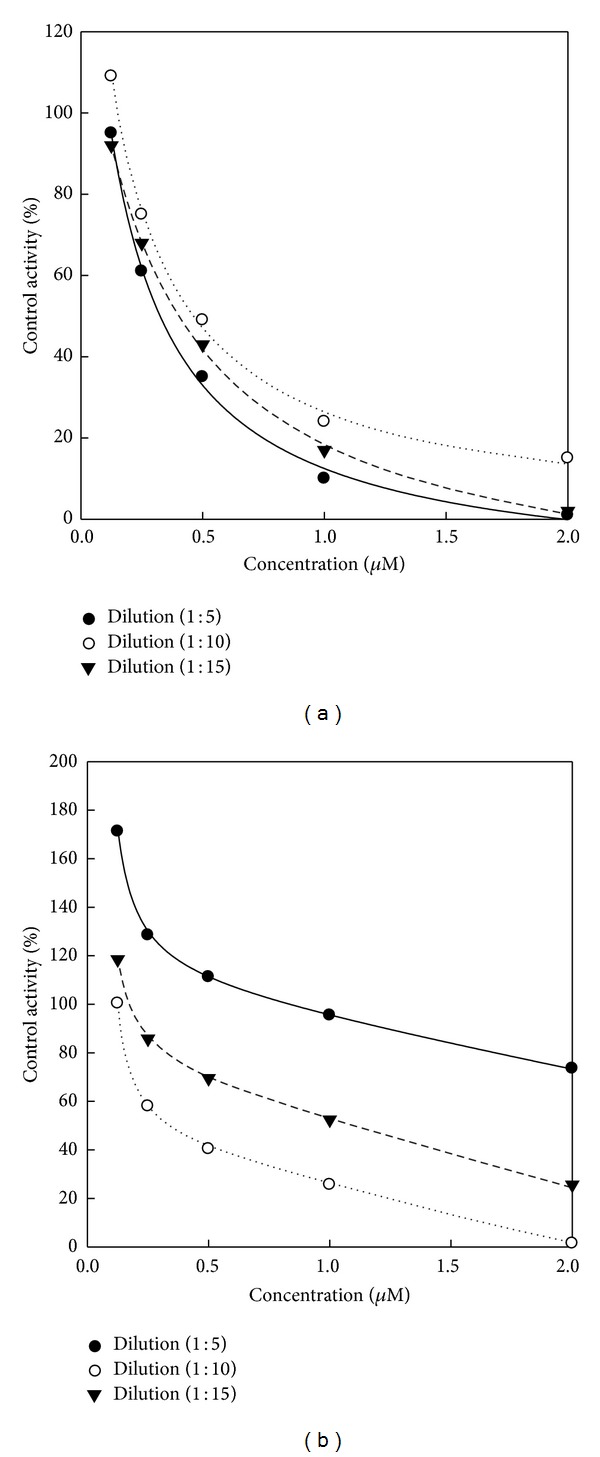
Concentration-inhibition curves for malathion in serum for quail in the presence of dilution 1 : 5, dilution 1 : 10, and dilution 1 : 15. Three preincubation conditions were used: long (4 h) (a) and short (1 h) (b). Experimental data (symbols) and model output (lines) are fitted by single rectangular equation of a hyperbola using a SigmaPlot 11.

Concentration-inhibition curves for malathion in the serum for quail in presence of dilution 1 : 5, dilution 1 : 10, and dilution 1 : 15 were found to be the highest reaction rates in dilution 1 : 10 for long time (4 h), whereas reducing reaction 10 rates in dilution 1 : 10 for short time (1 h) (Figure 8). The substrate specificity of the diluted esterases was investigated using two different kinds of substrates, PSA for the CbE activity and AcTChI for the ChE activity. Point estimates are reported in Table 2, along with the ratios of the different dilutions values. The somewhat smaller ratios at the higher concentrations (less than half at the IC_80_ compared to IC_20_) with dilution 1 : 10, but not dilution 1 : 15, suggest some saturation of the CbE in that range of the curve. Esterase activity of the enzyme was determined using PSA and AcTChI-esters containing a various thiol side chain length. As shown in Table 3, the highest *k*_cat_ value was observed with PSA-substrate (dilution 1 : 5), whereas AcTChI-substrate (dilution 1 : 15) showed the lowest *k*_cat_ value. However, the *K*_*m*_ value was the highest with PSA-substrate (dilution 1 : 15) and the lowest with AcTChI-substrate (dilution 1 : 10). Among the thiol-esters dilution 1 : 5 was observed to have the highest specificity constant (*k*_cat_/*K*_*m*_), and the *K*_*m*_ and *k*_cat_ values were 176 *μ*M and 16, 765 s^−1^, respectively, for PSA ester, while detected in dilution 1 : 15 the lowest specificity constant (*k*_cat_/*K*_*m*_), and the *K*_*m*_ and *k*_cat_ values were 943 *μ*M and 1154 s^−1^, respectively, for AcTChI ester.

## 4. Discussion

### 4.1. The Evaluation of Esterase Enzyme Dilution Assays

Two forms of esterase enzyme activities have been identified in mammalian blood. These are distinguished according to their substrate specificity [2]. In fact, CbE prefers PSA as substrate (at high concentrations) but ChE prefers AcTChI as substrate (at low concentrations) [26–29]. In this paper the author showed five dilutions to measure the activities of CbE and ChE in plasma, serum, and erythrocyte for quail, duck, and chick to reduce the turbidity of the blood samples and to ensure that the optical signal falls within the linear range of detection throughout the esterase enzyme activity measurement, and the enzyme dilution slows down the rate of spending of substrate, so providing an extended time window for statement of steady-state esterase enzyme kinetics [18, 30]. The results from this study show that dilution 1 : 10 had high esterases enzyme activity over different dilutions used and ranged between 172.3 and 590.3 nmol min^−1^ mL^−1^ for plasma, between 293.4 and 996.4 nmol min^−1^ mL^−1^ for erythrocyte, and between 180.6 and 763.5 nmol min^−1^ mL^−1^ for serum (Figures 1–3). A similar observation has been made by Quinn et al. [31], reporting that dilution 1 : 10 for plasma and erythrocyte had higher activity than dilution 1 : 25. These interesting results are also similar with finding of  [32], where they found it higher in the blood for ChE activity with dilution 1 : 10. The reason for this is probably due to adsorption changes occurring faster at dilution 1 : 10 than other dilutions [33]. Esterase activity in the blood was at the higher dilution ratio and declined faster than that of samples at lower dilution ratio. Linear regression of between CbE and ChE activities was observed in five dilutions samples for all birds tested with blood with exception of quail (Figure 5).

### 4.2. A Kinetic Model for Substrate and Dilution of Blood Samples under OP-Sufficient Conditions

The results of kinetic studies using thiol side esters for the esterase enzyme show that PSA for the CbE activity and AcTChI for the ChE activity (dilution 1 : 10) are the most suitable substrate for the enzyme kinetic. The somewhat smaller ratios at the higher concentrations (less than half at the IC_80_ compared to IC_20_) with dilution 1 : 10, but not dilution 1 : 15, suggest some saturation of the CbE in that range of the curve (Table 2). This is in agreement with the previous study [34]. In summary, this study used an* in vitro* assay to measure detoxification by CbE and ChE of a malathion compound (Table 3). This screening method could be useful for predicting the degree of esterase detoxification, and this information could be useful in assessment of animal variability due to differences in esterase activities [2]. Dilution 1 : 5 was observed to have the highest specificity constant (*k*_cat_/*K*_*m*_), and the *K*_*m*_ and *k*_cat_ values were 176 *μ*M and 16,765 s^−1^, respectively, for PSA ester, while dilution 1 : 15 had the lowest specificity constant (*k*_cat_/*K*_*m*_), and the *K*_*m*_ and *k*_cat_ values were 943 *μ*M and 1154 s^−1^, respectively, for AcTChI ester. For many of this inhibitor, these are the first data available for informing esterase enzyme mediated detoxification. Our findings indicate that detoxification patterns are chemical-specific and that the influence of known susceptibility factors such as CbE and ChE should not be generalized across this inhibitor [35].

## 5. Conclusions

In conclusion, this is the first paper that provided original data concerning an enzymological dilution characterization in the blood samples from birds used for human consumption. This paper succeed in establishing a gentle dilution in all three tested birds (quail, duck, and chick) and blood (plasma, serum, and erythrocyte); the dilution 1 : 10 recorded higher esterase enzyme activity than other dilutions (1 : 5, 1 : 15, 1 : 20, and 1 : 25). Furthermore, our results also pointed at the importance of estimating different kinetic dilutions effects prior to using in birds as biomarker tools of environmental exposure to anti-CbE or anti-ChE pesticides intoxication. Finally, in spite of this paper, further studies are required under different laboratories and different OP pesticide compounds in order to improve and to increase our knowledge about this very interesting enzyme as a potential biochemical marker for pesticide compounds.

## Figures and Tables

**Table 1:** Pearson correlation coefficient and *P* values between CbE and ChE in the blood for birds at different dilutions.

Dilution	Plasma			Erythrocyte			Serum		
	Quail	Duck	Chick	Quail	Duck	Chick	Quail	Duck	Chick
1 : 5	0.219	−0.111	−0.402	−0.648	−0.063	0.112	0.411	0.102	0.071
	(0.22)	(0.52)	(0.22)	(0.21)	(0.82)	(0.21)	(0.22)	(0.71)	(0.10)
1 : 10	−0.337	0.081	0.123	0.253	−0.580	−0.326	0.026	0.209	0.138
	(0.10)	(0.90)	(0.04)	(0.13)	(0.13)	(0.02)	(0.93)	(0.52)	(0. 06)
1 : 15	0.311	−0.179	−0.083	0.152	−0.451	−0.224	0.544	−0.091	0.417
	(0.31)	(0.32)	(0.21)	(0.54)	(0.12)	(0.74)	(0.04)	(0.92)	(0.09)
1 : 20	−0.245	0.162	0.242	0.352	−0.324	0.157	0.446	−0.216	0.429
	(0.74)	(0.13)	(0.12)	(0.43)	(0.24)	(0.55)	( 0.04)	(0.02)	(0.18)
1 : 25	0.221	0.205	0.134	−0.078	−0.121	−0.251	0.559	−0.075	0.317
	(0.11)	(0.89)	(0.43)	(0.13)	(0.42)	(0.65)	(0.32)	(0.08)	(0.12)

Values in the table are the *r* (*P* value).

**Table 2:** Malathion point estimates (IC_20_, IC_50_, and IC_80_; *μ*M) from concentration-inhibition curves in the presence of dilutions 1 : 5, 1 : 10, and 1 : 15. Two preincubation conditions were used: long (4 h) and short (1 h).

Preincubation condition	Slop	IC_20_ (*μ*M)	Slop	IC_50_ (*μ*M)	Slop	IC_80_ (*μ*M)
Long (4 h)						
Dilution (1 : 5)	0.22 ± 0.655^a^	3.15 ± 0.991^a^	0.67 ± 0.126	1.1 ± 0.432^a^	0.66 ± 0.718	1.1 ± 0.121
Dilution (1 : 10)	1.4 ± 0.122	0.50 ± 0.654	0.89 ± 0.117	0.78 ± 0.121	0.64 ± 0.611	1.2 ± 0.211
Dilution (1 : 15)	0.11 ± 0.418^b^	6.3 ± 1.2^b^	0.71 ± 0.112	0.98 ± 0.212	0.84 ± 0.801	0.83 ± 0.221
Short (1 h)						
Dilution (1 : 5)	0.34 ± 0.372^a^	2.1 ± 0.876^a^	0.29 ± 0.501^a^	2.4 ± 0.311^a^	0.37 ± 0.48^a^	1.9 ± 0.421
Dilution (1 : 10)	0.66 ± 0.931	1.1 ± 0.765	0.27 ± 0.145	2.6 ± 0.911	0.29 ± 0.18	2.4 ± 0.987
Dilution (1 : 15)	0.12 ± 0.583^b^	5.8 ± 1.1^b^	0.53 ± 0.196^b^	1.31 ± 0.876	0.24 ± 0.41^b^	2.9 ± 0.766

Values in the table are the mean ± SE of three individual serum esterase (CbE) for quail. For the measurement of IC_20_, IC_50_, and IC_80_, esterase enzyme was inhibited for 30 min at room temperature 25°C with appropriate concentrations for malathion compound. Then results were fitted with an exponential decay using SigmaPlot 11. ^a,b^denotes significant difference (analysis of variance (ANOVA), *P* < 0.05) between dilution 1 : 5 and dilution 1 : 15.

**Table 3:** Kinetic parameters for hydrolysis of esterase in various dilutions.

Preincubation condition	CbE			ChE		
	*K*_*m*_ (*μ*M)	*k*_cat_ (s^−1^)	*k*_cat_/*K*_*m*_ (s^−1^ *μ*M^−1^)	*K*_*m*_ (*μ*M)	*k*_cat_ (s^−1^)	*k*_cat_/*K*_*m*_ (s^−1^ *μ*M^−1^)
Dilution (1 : 5)	176 ± 15.3^a^	16,765 ± 865^a^	95.3^a^	265 ± 23.4^a^	2023 ± 232^a^	7.6^a^
Dilution (1 : 10)	543 ± 21.3	12,765 ± 932	23.5	234 ± 32.4	17,165 ± 654	73.4
Dilution (1 : 15)	753 ± 28.4	7653 ± 121^b^	10.2	943 ± 71.2	1154 ± 111	1.2

Values in the table are the mean ± SE of three individual serum esterase (CbE and ChE) for quail. For the measurement of *K*_*m*_ and *k*_cat_, esterase enzyme was inhibited for 30 min at room temperature 25°C with appropriate concentrations for malathion compound. Then results were fitted with an exponential decay using SigmaPlot 11. ^a,b^denotes significant difference (analysis of variance (ANOVA), *P* < 0.05) between dilution 1 : 5 and other dilutions.
